# Does place of birth influence endogenous hormone levels in Asian-American women?

**DOI:** 10.1038/sj.bjc.6600339

**Published:** 2002-07-15

**Authors:** R T Falk, T R Fears, R N Hoover, M C Pike, A H Wu, A M Y Nomura, L N Kolonel, D W West, R G Ziegler

**Affiliations:** Division of Cancer Epidemiology and Genetics, National Cancer Institute, Bethesda, Maryland, MD 20892, USA; Department of Preventive Medicine, University of Southern California, Keck School of Medicine, Los Angeles, California, CA 90033, USA; Epidemiology Program, Cancer Research Center of Hawaii, University of Hawaii, Honolulu, Hawaii, HI 96817, USA; Northern California Cancer Center, Union City, California, CA 94587, USA

**Keywords:** Asian-American, oestrogen, androgen, breast cancer, migration

## Abstract

In 1983–87, we conducted a population-based case–control study of breast cancer in Asian women living in California and Hawaii, in which migration history (a composite of the subject's place of birth, usual residence in Asia (urban/rural), length of time living in the West, and grandparents' place of birth) was associated with a six-fold risk gradient that paralleled the historical differences in incidence rates between the US and Asian countries. This provided the opportunity to determine whether endogenous hormones vary with migration history in Asian-American women. Plasma obtained from 316 premenopausal and 177 naturally premenopausal study controls was measured for levels of estrone (E1), estradiol (E2), estrone sulphate (E1S), androstenedione (A), testosterone (T), dehydroepiandrosterone (DHEA), dehydroepiandrosterone sulphate (DHEAS), progesterone (PROG) and sex hormone-binding globulin (SHBG). Levels of the oestrogens and sex hormone-binding globulin did not differ significantly between Asian- and Western-born women, although among premenopausal women, those least westernised had the lowest levels of E1, E2, and E1S. Androgen levels, particularly DHEA, were lower in women born in the West. Among premenopausal women, age-adjusted geometric mean levels of DHEA were 16.5 and 13.8 nmol l^−1^ in Asian- and Western-born women respectively; in postmenopausal women these values were 11.8 and 9.2 nmol l^−1^, (*P*<0.001) respectively. Among postmenopausal women, androgens tended to be highest among the least westernised women and declined as the degree of westernisation increased. Our findings suggest that aspects of hormone metabolism play a role in population differences in breast cancer incidence.

*British Journal of Cancer* (2002) **87**, 54–60. doi:10.1038/sj.bjc.6600339 www.bjcancer.com

© 2002 Cancer Research UK

## 

Breast cancer incidence rates among US and Western European women have been nearly six-fold higher than those in Asia. Since the 1970's studies of endogenous hormone levels in healthy Asian and Caucasian women have produced varied results. Most (MacMahon *et al*, 1974, 1980; Moore *et al*, 1983; Trichopoulos *et al*, 1984; Goldin *et al*, 1986; Bernstein *et al*, 1990; Shimizu *et al*, 1990; Key *et al*, 1990), but not all (Bulbrook *et al*, 1976, Hayward *et al*, 1978; Goodman *et al*, 1988; Porbst-Hensch, 2000) found higher oestrogens in Caucasian women. In the few studies that measured androgens (Bulbrook *et al*, 1967; Wang *et al*, 1976; Hayward *et al*, 1978), levels were higher in Caucasian women. The comparisons mainly concerned hormone levels in Asians living in the East with those of Caucasian women in the West (see Discussion) though a few studied Asian and Caucasian women living in the same region in the West (Goldin *et al*, 1986; Goodman *et al*, 1988; Probst-Hensch *et al*, 2000). In only two studies were women selected as representative of a defined population (Bernstein *et al*, 1990; Probst-Hensch *et al*, 2000).

Different genetic susceptibilities alone are an unlikely explanation for international differences in breast cancer incidence, since rates among successive generations of Asian migrants to the West approach those of Caucasians (Buell, 1973). More recently, the magnitude of these differences has declined as a Western lifestyle has spread in Asia (Coleman *et al*, 1993). In 1983–87, we conducted a population-based case–control study of incident breast cancer in Asian-American women in which women born in the West had an approximate two-fold risk compared to those who had migrated from Asia and a six-fold spread in risk when additional aspects of migration history were considered (Ziegler *et al*, 1993). This presented an opportunity to determine whether endogenous hormones vary according to migration history among women of Asian descent living in the US. To this end, we measured the following steroid sex hormones and binding proteins in the plasma of study controls: E1, E2, E1S, A, T, DHEA, DHEAS, PROG, and SHBG.

## MATERIALS AND METHODS

### Study population

The design of this population-based study has been reported in detail elsewhere (Ziegler *et al*, 1993). Briefly, women of Chinese, Japanese or Filipino descent, aged 20–55 years, and diagnosed with histologically-confirmed incident breast cancer between April 1983 and June 1987 in the San-Francisco–Oakland Metropolitan Statistical Area (MSA), the Los Angeles MSA and Oahu, Hawaii were identified. Population controls were frequency-matched to cases in their region by ethnicity and year of birth, within 5 years. Controls from San Francisco–Oakland and Los Angeles were obtained by random-digit dialling; in Hawaii, controls were obtained through the Health Surveillance Program which annually samples 2% of households in the state. Where possible, a 2 : 1 ratio of controls to cases was obtained. Participation rates among eligible controls were as follows: Chinese 72%, Japanese 78%, and Filipino 73%.

### Study methods

Participants were interviewed at home by trained interviewers in the subject's choice of language: Chinese, Japanese or English. The questionnaire obtained information on the subject's residential history, place of birth of parents and grandparents, reproductive and menstrual history, medical history and cancer in family members, anthropometry and diet. Women were asked to identify each country (and state, if in the US) in which they had lived for at least 1 year, and for each, the length of time at that residence and whether the community was urban (city or suburb) or rural (town, village or rural area). Countries of birth considered part of the ‘East’ included: China, Taiwan, Hong Kong, Macao, Japan, the Philippines, Southeast Asia, the Malaysian Peninsula, Singapore, India and countries in the southwest Pacific Ocean except Australia and New Zealand. ‘West’ included countries in North America, Western Europe, Central Europe, the former USSR, Australia and New Zealand.

Among menstruating women, the blood draw was scheduled to coincide with the mid-luteal phase, estimated to occur between days 19 and 26 of the menstrual cycle. To facilitate this, women were telephoned to determine the start date of their current menstrual cycle and estimate the start of their next cycle. After the blood draw, women were instructed to return a postcard indicating the starting date of their next menses. Women were considered postmenopausal if their last menses occurred more than 1 year prior to blood draw.

**Figure 1:**
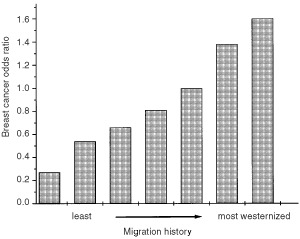
Breast cancer odds ratios for Asian-American women according to migration history. Migration categories are from left to right as follows: born in the East, rural in East, living in West <8 years; born in the East, urban in East, living in West <8 years; born in the East, rural in East, living in West 8+ years; born in the East, urban in East, living in West 8+ years; born in the West, all grandparents born in the East; born in the West, one or two grandparents born West; born in the West, at least three grandparents born in the West.

In the case-control study, a composite measure of several aspects of migration history demonstrated a pattern of breast cancer risk that paralleled the six-fold difference in incidence rates between the US and Asian countries (Ziegler *et al*, 1993). Figure 1

shows the ORs from the full case–control study according to this measure with bars from left to right corresponding to the risks for the least to the most westernised. All ORs were calculated in relation to women born in the West whose grandparents were born in the East. Thus, for example, the leftmost bar is the risk for those born in the East in a rural environment and living in the US for fewer than 8 years, while the rightmost bar is the risk for women born in the West with three or more grandparents also born in the West. For the subset of cases and controls participating in the blood collection component, risks according to these categories of migration history showed a similar pattern, with ORs increasing from 0.4 to 1.2 (results not shown). In this study, westernisation refers to this composite variable, with the exception being that the two most westernised categories (born in the West with one or two grandparents born in the West, and born in the West with at least three grandparents born in the West; see legend to Figure 1) were combined because of the small number of women in each category.

### Laboratory methods

Fasting morning blood was drawn from participants into EDTA tubes which were placed on ice and separated by centrifugation within 3 h. Samples were then stored at −70°C until being sent to a repository for long-term storage. Assays were conducted at Quest Diagnostics, San Juan Capistrano, CA, USA, where E1, E2, T, A, DHEA and PROG were measured by updated radioimmunoassays (RIA) for the specific analyte after extraction from plasma with ethyl acetate : hexane and purification by celite chromatography (Quest Diagnostics Clinical Studies Center Reference Manual, 1997; England *et al*, 1974; Judd *et al*, 1976; Abraham *et al*, 1971, 1975, 1976; Gives, 1976; Korth-Schutz *et al*, 1978; Kinouchi *et al*, 1973; Kirschner and Bardin, 1972; Rao and Moore, 1977; DePeretti and Forest, 1976). For E1S, unconjugated estrone was extracted by ethyl acetate : hexane, hydrolyzed overnight with the resultant E1 extracted and measured by RIA (Wright *et al*, 1978; Loriaux *et al*, 1971). DHEAS was measured directly by RIA after dilution of plasma with assay buffer (DePeretti and Forest, 1976; Buster and Abraham, 1972). SHBG was measured by RIA (DSL, Webster, TX, USA). Per cent free E2 was calculated by the method of Södergard *et al*, 1982 and Vermeulen *et al* (1999). Assay sensitivities were as follows: E2, 7.4 pmol l^−1^; E1, 36.7 pmol l^−1^; E1S, 142.7 pmol l^−1^; T, 0.07 nmol l^−1^; A, 0.1 nmol l^−1^; DHEA, 4.6 nmol l^−1^; DHEAS, 0.14 μmol l^−1^; SHBG 5.0 nmol l^−1^; PROG, 0.2 nmol l^−1^. Progesterone values were obtained for the purpose of clarifying the time of menstrual cycle and are not reported here.

The intra- and interassay CVs obtained from blinded quality control samples added to our study batches were as follows: E2, 19% and 17%; E1, 4% and 9%; E1S, 8% and 10%; DHEA, 6% and 3%; DHEAS, 5% and 8%; A, 8% and 7%; T, 6% and 6%; SHBG, 4% and 4%; and PROG, 14% and 10%; respectively. The relatively poor performance of the E2 assay was due to a quality control pool with values near the assay limit of detection. Without this specimen, the intra- and interassay CVs for E2 were 6 and 6%, respectively.

### Statistical methods

Hormone measurements were analysed on the natural logarithmic scale to reduce the dependence of the variance on the mean response. Assay results for premenopausal and postmenopausal women were analysed separately. Geometric mean hormone levels were obtained using standard analysis of covariance techniques, adjusting for age at blood collection (continuous) and ethnicity. Analyses of luteal phase bloods included additional adjustment for the number of days between blood draw and subsequent menses (continuous, modeled with linear and quadratic terms). Differences in the distributions of menstrual, reproductive and demographic characteristics according to place of birth were tested using Chi-square statistics. Tests of trends in hormones across levels of migration history were obtained by scoring the categories and considering it a continuous variable in standard regression models. Tests for hormone differences between specific levels of the migration variable were obtained from appropriate contrasts. Analyses were conducted using SYSTAT software (Wilkinson, 1986).

## RESULTS

A total of 966 controls participated in the interview study. Of these, fasting morning blood samples were obtained from 568 women, including 133 Chinese (46% participating), 276 Japanese (70% participating) and 159 Filipino (56% participating) women. Participation in the blood draw was higher among more westernised women. Of those born in the East, 50% agreed to a blood collection, whereas close to 70% of those born in the West agreed to donate blood. This difference in blood collection participation rate by place of birth was similar for the three ethnic groups. Compared to women for whom we did not have a blood sample, participants were slightly taller (*P*<0.05), had a higher BMI (*P*<0.001), and were younger at menarche (*P*<0.001), but were similar with respect to age and other reproductive characteristics.

Of the 568 participating controls, bloods from 61 (11%) reporting exogenous hormone use were not assayed. Hormone measurements were obtained for a total of 507 women, including: 317 premenopausal and 117 naturally postmenopausal women, 20 women with surgical removal of both ovaries, 11 women with surgical removal of one ovary, 32 women with surgical removal of the uterus only, eight with cessation of menses within 6–12 months of blood draw and two with unknown menopausal status. For this study, we included only premenopausal or naturally postmenopausal women not currently using exogenous hormones. Excluding one premenopausal woman whose place of birth was not known, this includes a total of 316 premenopausal and 117 naturally postmenopausal women.

### Demographic and hormone-related factors

Selected demographic, anthropometric and reproduction-related factors are shown in Table 1

**Table 1:**
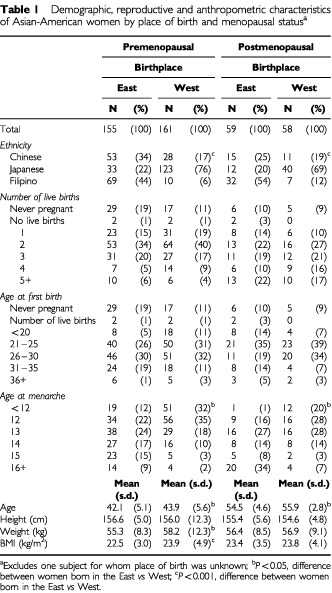
Demographic, reproductive and anthropometric characteristics of Asian-American women by place of birth and menopausal status^a^

according to place of birth. In both pre- and postmenopausal women, the ethnic distribution differed based on place of birth, with a majority of those born in the East being Filipino, and most born in the West being of Japanese descent. Neither the number of live births or age at first birth differed based on place of birth, but Western-born women were significantly younger at the start of menarche than Asian-born participants. Women born in the West were slightly older at blood draw, and among premenopausal women, those born in the West weighed more than those born in the East.

### Hormone levels

#### Premenopausal women

Blood from only 202 of the 316 premenopausal women (64%) was considered as drawn in the luteal phase, having been obtained between days 19–26 of the menstrual cycle or having evidence of ovulation with progesterone values above 9.54 nmol l^−1^. Analyses adjusted for age and ethnicity were conducted with all premenopausal women, and separately for the subset of luteal phase women with additional adjustment for the number of days from blood draw to start of menses (Table 2

**Table 2:**
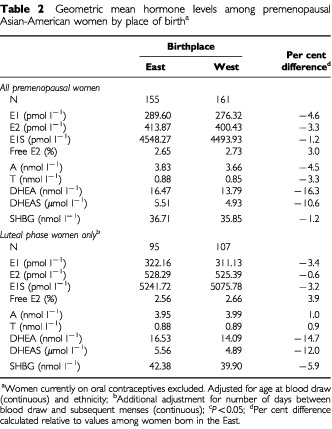
Geometric mean hormone levels among premenopausal Asian-American women by place of birth^a^

). Among the entire premenopausal group, no significant differences with regard to place of birth were observed for the oestrogens, with levels of E1, E2, E1S and per cent free E2 each less than 5% lower in Western-born women. For the androgens, differences in A and T were less than 5% and not significant, but DHEA and DHEAS were more than 10% lower in Western-born women. For SHBG, the difference based on place of birth was marginal, but values were significantly lower in Filipino women than in women of Chinese or Japanese descent. Analyses restricted to luteal phase women showed similar patterns, with marginal differences in the oestrogens based on place of birth, and levels of DHEA and DHEAS more than 10% lower in women born in the West. Adjusting for height, weight, BMI, parity or ages at menarche and first live birth did not alter the pattern of these findings (results not shown).

**Figure 2:**
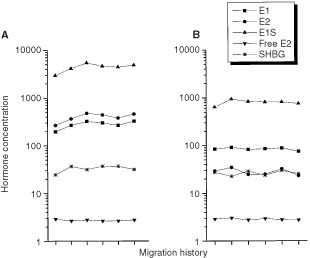
Oestrogen and SHBG levels in Asian-American women according to migration history. (**A**) and (**B**) correspond to levels in pre- and postmenopausal women respectively. Migration categories are from left to right as follows (number of pre- and post-menopausal women in parentheses): born in the East, rural in East, living in West <8 years (10, 5); born in the East, urban in East, living in the West <8 years (37, 9); born in the East, rural in East, living in West 8+ years (26, 11); born in the East, urban in East, living in the West 8+ years (55, 22); born in the West, all grandparents born in the East (137, 56); born in the West, at least one grandparent born in the West (24,2). Excluded are 27 pre-, 12 postmenopausal women missing information on type of residence in the East, number of years living in the West or place of birth of grandparents. Plasma levels of E1, E2 E1S are in pmol l^−1^; SHBG is in nmol l^−1^; free E2 is expressed as a per cent.

**Figure 3:**
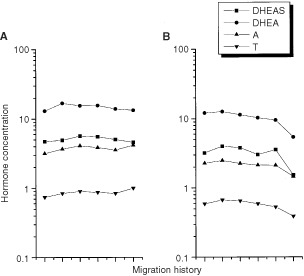
Androgen levels in Asian-American women according to migration history. (**A**) and (**B**) correspond to levels in pre- and postmenopausal women respectively. Migration categories are from left to right as follows (number of pre- and post-menopausal women in paren.): born in the East, rural in the East, living in the West <8 years (10, 5); born in the East, urban in the East, living in the West <8 years (37, 9); born in the East, rural in the East, living in the West 8+ years (26, 11); born in the East, urban in the East, living in the West 8+ years (55, 22); born in the West, all grandparents born in the East (137, 56); born in the West, at least one grandparent born in the West (24,2). Excluded are 27 pre-, 12 postmenopausal women missing information on type of residence in the East, number of years living in the West or place of birth of grandparents. Plasma levels of DHEA, A and T are in nmol l^−1^; for DHEAS, the level is μmol l^−1^.

Plots of mean hormone levels according to migration history are shown for premenopausal women in Figure 2A

(oestrogens and SHBG) and Figure 3A

(androgens). In interpreting these plots, we note that few women were in the extreme categories of migration history (10 and 24 women in the least and most westernised categories, respectively). No trends were observed in the oestrogens according to migration history, but E1, E2 and E1S tended to be lowest among the least westernised women (the left most symbol in Figure 2A). In particular, E2 values in these women were more than 50% lower than values in the most westernised women (*P*=0.11, difference in E2 between the extreme migration categories). Androgens did not trend with migration history (Figure 3A). Hormone patterns did not change with adjustment for weight, height, BMI, parity or ages at menarche and first birth (results not shown).

#### Postmenopausal women

After adjustment for age and ethnicity, differences in the oestrogens based on place of birth were less than 5% and not significant (Table 3

**Table 3:**
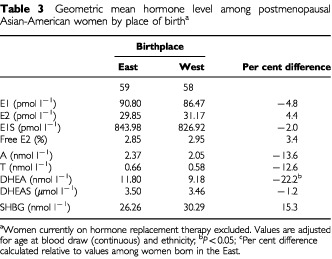
Geometric mean hormone level among postmenopausal Asian-American women by place of birth^a^

). The androgens tended to be lower in Western-born women, with differences of more than 10% observed for A and T. The difference in DHEA was significant, with levels more than 20% lower in Western-born women. SHBG was higher in Western-born women, but this difference was not significant. As observed in premenopausal women, levels of SHBG were significantly lower in Filipino women compared to women of Chinese or Japanese descent. Adjustment for weight, height, BMI, parity, or ages at menarche and first birth did not change these findings (results not shown).

Plots of oestrogen and androgen levels according to migration history are shown in Figures 2B and 3B, respectively, where the number of postmenopausal women in the extreme categories was very small (five and two, respectively in the least and most westernized category). No trends according to migration history were observed for the oestrogens in postmenopausal women. Androgen levels tended to decline as the degree of westernisation increased but only the trend for DHEA was suggestive (*P*, trend=0.06).

## DISCUSSION

In an effort to explain the international differences in breast cancer incidence rates, a number of investigators have compared hormone levels in healthy Asian and Caucasian women, with most focusing on differences in oestrogen levels (MacMahon *et al*, 1974; Moore *et al*, 1983; Trichopoulos *et al*, 1984; Goldin *et al*, 1986; Goodman *et al*, 1988; Bernstein *et al*, 1990; Shimizu *et al*, 1990; Key *et al*, 1990; Probst-Hensch *et al*, 2000) and a few examining androgens or androgen precursors (Bulbrook *et al*, 1967; Wang *et al*, 1976; Hayward *et al*, 1978). However, these studies could not distinguish the role of genetic differences from the effects of an Asian lifestyle on endogenous hormone levels. By limiting this study to women of Asian descent in the US, we sought to isolate the influence of lifestyle alone. Our main finding was that androgens, particularly DHEA, were lower in Western-born Asian women; this was true in both pre- and postmenopausal women. Moreover, in postmenopausal women, the androgens tended to be highest among the least westernised women and declined as the degree of westernisation increased. For the oestrogens, levels between Asian- and Western-born women were not significantly different in pre- or postmenopausal women, and no clear patterns according to degree of westernisation were observed. In premenopausal women however, oestrogens were lowest in the least westernised women. To the extent that hormone levels among women in our extreme categories of westernisation reflect those of the Asian and Caucasian women that have historically been studied, the small number – 15 and 26 women in the least and most westernised groups, respectively – provided limited power to explore possible differences between them.

The extensive literature on ethnic differences in endogenous oestrogens is mixed. A number of factors may have contributed to this inconsistency, the most important being the dramatic improvement in assay methodology over the time these studies were conducted. Before the 1990s, little attention had been paid to the performance of steroid hormone assays within the normal range, and problems with assay precision and accuracy could have obscured small but consequential differences. Since then, a number of studies have assessed laboratory assay reproducibility (Cauley *et al*, 1991; Potischman *et al*, 1994; Hankinson *et al*, 1994; Toniolo *et al*, 1994; Gail *et al*, 1996; Fears *et al*, 2000; Falk *et al*, 1999). Most demonstrate that variability is not negligible from one batch to the next even in the same laboratory, and rigorous quality control efforts must be implemented to ensure the reliability of results. Other reasons for inconsistencies across studies may relate to specimen collection, with samples ranging from a single urine or blood draw to multiple collections over a 24 h period. Finally, comparisons across studies are complicated by study designs in which women were not representative of the target populations (Bulbrook *et al*, 1967, 1976; MacMahon *et al*, 1974, 1980; Wang *et al*, 1976; Hayward *et al*, 1978; Moore *et al*, 1983; Trichopoulos *et al*, 1984; Goldin *et al*, 1986; Goodman *et al*, 1988; Shimizu *et al*, 1990; Key *et al*, 1990).

In light of these issues, we consider recent results from studies conducted in defined populations and using standardised assay methods to be the most useful comparisons for our study. These include findings from controls participating in a population-based case-control study of breast cancer in the US and China (Bernstein *et al*, 1990), and hormone comparisons between Japanese-Americans and US Caucasians participating in the Multiethnic Cohort (Probst-Hensch *et al*, 2000). These studies showed oestrogens were higher in Caucasians than Asian women living in China, but similar to, if not slightly lower than levels in Japanese-Americans.

Concern that a single specimen may not correctly characterise an individual's hormone levels have long hampered efforts to study endogenous hormones in premenopausal women. Indeed, studies with blood collected between 1 and 3 years apart have demonstrated that levels of androgens, but not oestrogens, tend to be consistent from one year to the next (Muti *et al*, 1996; Michaud *et al*, 1999; Hankinson *et al*, 1995). In particular, the reproducibility of E2 was quite poor. By restricting analyses to blood collected between 4–10 days prior to the next menses, one study observed a dramatic improvement in the intraclass correlation coefficient (Michaud *et al*, 1999), suggesting that a single, carefully scheduled blood collection may be sufficient to characterise luteal phase oestrogens. We found that compared to the entire premenopausal group, estrogen levels in women known to be in mid-luteal phase were higher overall, but differences between those born in the East *vs* the West were similar in magnitude and direction.

The finding of lower androgen levels in our Western-born Asian women is provocative yet at variance with a few early studies that found lower levels in Asian women compared to Caucasians. As with the oestrogens, changes in androgen assays limit our comparisons with these early investigations, and recent studies of endogenous androgens are lacking. Since the patterns of results for T, A, DHEA and DHEAS were very similar across our indicator of westernisation, and the absolute levels of these hormones were comparable to values reported in a recent study using the same laboratory and assay procedures (Hankinson *et al*, 1998), we are reasonably confident of the assay methods and results. Nevertheless, interpretation of our findings is complicated because participation in the blood collection component of the study was influenced by a woman's degree of westernisation. Among Japanese and Chinese controls, those born in the West were more likely to contribute blood than those born in the East (for Japanese women, participation rates were 72 and 63% in women born in the West *vs* the East; for Chinese, these figures were 66 and 38%, respectively).

The study of migrant groups such as this one offers an opportunity to explore the role of hormonal factors that have figured prominently in hypotheses attempting to explain large international differences in breast cancer risk. Unfortunately, even though this is the largest study of Asian women in the United States, the small number of women in the most extreme categories of migration history prevented us from clarifying much about changes in oestrogens with westernisation. The finding of a decline in androgens with increasing westernisation is new, provocative, and consistent with early experimental findings of antineoplastic effects of androgens on oestrogen-induced carcinogenesis (Schwartz, 1979, Schwartz and Tannen, 1981). By contrast, recent human studies have suggested a hazardous role for androgens (Berrino *et al*, 1996; Dorgan *et al*, 1996, 1997; Zeleniuch-Jacquotte *et al*, 1997), perhaps linked to their role as precursors for oestrogen synthesis. Relatively little research has focused on a potential dual role of androgens in breast neoplasia, although one study suggests that DHEA may have differing effects depending on estradiol levels; specifically, that DHEA stimulates cell growth in an estradiol-free medium and inhibits growth in the presence of estradiol (Bocuzzi *et al*, 1992). Future studies exploring the hormonal mechanisms that may underlie breast cancer risk factors should focus their efforts on a large spectrum of hormones including estrogens, androgens and their metabolites.
